# Phospholipid Nanoparticle Resuscitation Preserves Neuronal Integrity and Cognitive Recovery Without Exacerbating Neuroinflammation Following Hemorrhagic Shock-Induced Clinical Death

**DOI:** 10.3390/biomedicines14051020

**Published:** 2026-04-30

**Authors:** Philemon Shallie, Nathan Carpenter, Othman Sheikh Hussein, Harshini Kumaresan, Danielle Kinsey, Oluwadamilola Shallie, Gelilla Daniel, Gracy Rosario, Michael Moncure, Cuthbert O. Simpkins

**Affiliations:** 1 Department of Surgery, School of Medicine, University of Missouri, Kansas City, MO 64108, USA; njc6f7@umkc.edu (N.C.); ost2p@umkc.edu (O.S.H.); hkumaresan@umkc.edu (H.K.); djk994@umkc.edu (D.K.); gelilladaniel@umkc.edu (G.D.); gxrf7c@umkc.edu (G.R.); moncurem@umkc.edu (M.M.); 2 Department of Human Anatomy, University of Saint Mary, Leavenworth, KS 66048, USA; damie.shallie@stmary.edu; 3 Department of Surgery, University Health Truman Medical Center, Kansas City, MO 64108, USA

**Keywords:** hemorrhagic shock, ischemia–reperfusion injury, nanoparticle resuscitation, brain lesions, neuroprotection

## Abstract

**Background/Objectives**: Severe hemorrhagic shock progressing to clinical death remains a major cause of mortality and long-term neurological morbidity despite advances in trauma care. While current resuscitation strategies restore circulation, their ability to preserve brain structure and function following global ischemia–reperfusion injury remains limited. Hemorrhagic shock induces widespread neuronal vulnerability, particularly within the hippocampus and prefrontal cortex, contributing to persistent cognitive and behavioral deficits among survivors. **Methods:** Using a rat model of hemorrhagic shock-induced clinical death, we evaluated whether resuscitation with VBI-1, a phospholipid nanoparticle-based colloid, supports neurological recovery compared with whole blood-based resuscitation. Animals underwent controlled exsanguination to the point of clinical death, followed by rapid intra-arterial reanimation with either shed whole blood or VBI-1. Two phases of study were performed: histological evaluation of tissues 12 h after resuscitation and, in a separate cohort of animals, longitudinal behavioral recovery over 30 days. Histology focused on evaluating neuronal integrity in the hippocampal CA1 region and prefrontal cortex, neuronal functional status, and microglial responses. Sex was analyzed as a biological variable. **Results:** Resuscitation with VBI-1 is associated with sustained behavioral recovery, with pronounced sex-dependent effects favoring females during the subacute-to-chronic recovery phase. VBI-1 preserved neuronal density, laminar organization, and neuronal functional integrity in ischemia-vulnerable brain regions. This, and neuronal preservation, correlated with hippocampal-dependent working memory performance. Importantly, resuscitation with VBI-1 did not increase microglial density, coverage, or spatial organization, exacerbating the neuroinflammatory burden. **Conclusions:** These findings demonstrate that phospholipid nanoparticle-based resuscitation confers meaningful neurological recovery following profound circulatory collapse, highlighting the importance of evaluating resuscitation agents based on long-term brain outcomes.

## 1. Introduction

Trauma is a leading cause of mortality worldwide, resulting in more than four million deaths annually. Uncontrolled hemorrhage is the most common cause of preventable death across civilian trauma systems, prehospital care, and military settings [1,2]. Epidemiological analyses in North America underscore the importance of early hemorrhage control and timely access to definitive care, highlighting hemorrhage as a time-sensitive yet addressable contributor to trauma mortality [3]. Despite advances in damage-control surgery, evacuation logistics, and transfusion-based resuscitation protocols, outcomes following severe hemorrhagic shock remain poor, particularly when circulatory collapse progresses to clinical death. Globally, trauma remains a neglected disease of modern society, with limited progress in reducing subsequent organ injury and long-term morbidity among survivors [4].

Long-term morbidity remains substantial among patients who survive the initial hemorrhagic insult and resuscitation. Neurological dysfunction, including cognitive impairment, affective disturbances, and executive dysfunction, significantly contributes to disability, reduced quality of life, sustained healthcare burden, and delayed mortality in both civilian trauma survivors and wounded service members [5,6,7]. These outcomes highlight a critical gap in trauma care: survival does not equate to recovery, and preservation of neurological function remains an unmet clinical priority.

Severe hemorrhagic shock is increasingly recognized as a systemic ischemia–reperfusion syndrome with significant consequences for the central nervous system. Hypovolemia, clinical death, and the reanimation process contribute to a cascade of pathophysiological events, including cerebral hypoperfusion, mitochondrial dysfunction, oxidative stress, endothelial injury, and neuroinflammation, which collectively drive secondary brain injury [5,8,9]. Clinical and experimental studies have documented a wide range of neurological sequelae following profound hypovolemia and reperfusion, such as global cerebral ischemia, hemorrhagic encephalopathy, cerebral edema, ischemic stroke, cognitive decline, depression, anxiety, and post-traumatic stress disorder [6,7]. At the cellular level, ischemia–reperfusion injury induces excitotoxicity, blood–brain barrier disruption, lipid peroxidation, and inflammatory amplification within vulnerable neuronal populations [10,11]. These processes contribute to delayed neuronal degeneration and persistent neurological dysfunction long after apparent hemodynamic stabilization.

Current resuscitation strategies, including crystalloids, colloids, 1:1:1 transfusion, and whole blood, are limited in their ability to mitigate the neurobiological consequences of hemorrhagic shock. Although these approaches partially correct hypovolemia and restore perfusion pressure, they do not adequately address the cellular injury cascades triggered by global ischemia and reperfusion. Additionally, blood-based resuscitation and plasma products can exacerbate oxidative stress, promote lipid peroxidation, and increase free radical generation, intensifying reperfusion injury in vulnerable organs such as the brain [12,13,14]. Consequently, patients who initially survive hemorrhagic shock may later develop multiple organ dysfunction syndrome or persistent neurological deficits driven by secondary injury mechanisms rather than ongoing hypovolemia [15,16].

These limitations are especially apparent in cases of catastrophic hemorrhage, such as high-energy civilian trauma, prolonged prehospital transport, and battlefield injury, where massive blood loss can result in circulatory collapse or death. In these scenarios, no currently available resuscitation fluid reliably restores circulation while also protecting the brain and other vital organs from ischemia–reperfusion injury. This gap underscores the need for next-generation resuscitation strategies that go beyond volume replacement to actively preserve cellular and organ integrity.

An increasing body of evidence indicates that phospholipids function not only as structural components of cellular membranes but also as active mediators of neuronal survival and repair following injury. Phospholipid-based therapeutics, particularly those enriched with choline-containing head groups, have been shown to stabilize neuronal membranes, reduce lipid peroxidation, and preserve mitochondrial integrity under oxidative stress and ischemia–reperfusion injury. Among these, ganglioside GM1 has attracted significant attention for its neurotrophic and neuroprotective properties, including modulation of calcium homeostasis, enhancement of synaptic plasticity, and promotion of neuronal survival pathways. Recent translational studies have extended these findings to clinical contexts, such as a phase I clinical trial demonstrating the safety and tolerability of intravenous liposomal GM1 in patients with Parkinson’s disease [17]. In addition, targeted nanoparticle systems have been developed to enhance the delivery of neurotrophic agents across the blood–brain barrier; for example, OX26-conjugated ganglioside liposomes have been used to deliver CDP-choline in experimental stroke models, resulting in improved post-ischemic neuronal survival and functional recovery [18]. More broadly, choline-containing phospholipids have been extensively reviewed as modulators of the neurovascular unit, influencing membrane repair, synaptic signaling, and neuroinflammatory responses across various neurological disorders [19]. Collectively, these studies establish phospholipids as bioactive mediators that stabilize neuronal membranes, modulate oxidative injury, and promote functional recovery after central nervous system insults. To address this unmet need, we developed VBI-1, a phospholipid nanoparticle-based colloid designed to restore intravascular volume while attenuating oxidative and reperfusion-mediated tissue injury. In prior work, VBI-1 enabled successful reanimation following hemorrhagic shock-induced clinical death, restored hemodynamic stability, improved organ perfusion, and significantly reduced oxidative DNA damage compared with whole-blood resuscitation [20]. These findings identified VBI-1 as a promising resuscitation strategy with potential applicability across civilian emergency care, trauma surgery, and military medicine. Notably, unlike prior phospholipid-based approaches that primarily function as drug delivery vehicles or chronic neurotherapeutics, VBI-1 represents a distinct paradigm in which phospholipid nanoparticles are deployed as a systemic resuscitation fluid with intrinsic bioactive properties during acute global ischemia.

However, whether the systemic benefits of VBI-1 result in durable neurological protection and functional recovery remains unknown. The brain, particularly the hippocampal CA1 region and prefrontal cortex, is highly vulnerable to global ischemia and reperfusion. Delayed neuronal degeneration in these regions is strongly associated with impairments in memory, executive function, and affective regulation [8,21,22]. Recent clinical and imaging studies further implicate hippocampal subfield-specific structural and microstructural alterations in the development of cognitive impairment following vascular and ischemic insults [23]. Despite increasing recognition of this vulnerability, effective neuroprotective strategies during hemorrhagic shock and reanimation remain limited [24].

In this study, the Femoral Artery Catheterization and Reanimation Technique (FACART) was used to model hemorrhagic shock severe enough to induce clinical death, defined by a reduction in mean arterial pressure below 15 mmHg and cessation of spontaneous respiration. Clinical death in this model is defined by profound hypotension (MAP < 15 mmHg), cessation of spontaneous respiration, and loss of palpable pulses, physiological features that parallel terminal circulatory collapse observed in severe hemorrhagic shock and cardiac arrest. This model recapitulates a state of global ischemia followed by reperfusion, providing a translationally relevant platform for studying post-resuscitation brain injury. Animals were reanimated when spontaneous respiration resumed through rapid intra-arterial infusion of shed whole blood or VBI-1 and were monitored for 30 days. Behavioral recovery, hippocampal-dependent working memory, prefrontal cortical integrity, hippocampal CA1 neuronal survival, neuronal functional integrity as indicated by NeuroTrace labeling, and neuroinflammatory burden were assessed, with sex included as a biological variable.

It was hypothesized that VBI-1 would promote superior neurological recovery following reanimation from clinical death by preserving neuronal structure and function while avoiding exacerbation of post-ischemic neuroinflammation. Specifically, it was predicted that VBI-1 would limit delayed neuronal loss in ischemia-vulnerable regions such as the hippocampal CA1 and prefrontal cortex, preserve neuronal metabolic and translational capacity, and support sustained behavioral and cognitive recovery during the subacute-to-chronic phase after injury. By integrating longitudinal behavioral outcomes with multilevel histological and cellular analyses, this study aims to redefine success after hemorrhagic shock, shifting the focus from survival alone to the preservation of neurological function and long-term recovery.

## 2. Materials and Methods

### 2.1. Ethical Approval

The animal experiments were conducted in accordance with the “Guide for the Care and Use of Laboratory Animals,” published by the National Academy Press [25]. Ethics approval was obtained from the Institutional Animal Care and Use Committee (IACUC) at the University of Missouri, Kansas City, under Protocol 2108.

### 2.2. Development of the Phospholipid Nanoparticle (VBI-1)

VBI-1 is a phospholipid nanoparticle-based colloidal formulation developed as a multifunctional resuscitation fluid intended to restore intravascular volume while modulating nitric oxide (NO) bioavailability during ischemia–reperfusion. The formulation contains lecithin-derived phospholipids that, upon emulsification, self-assemble into nanoscale micellar nanoparticles with a hydrophobic soybean oil core stabilized by a phospholipid monolayer. In this structure, phospholipid acyl chains are oriented toward the hydrophobic core, while the polar head groups interact with the aqueous phase, forming a dispersed intravascular hydrophobic compartment. This organization facilitates preferential partitioning of the lipophilic NO molecule into the nanoparticle core and lipid domains, thereby reducing pathological NO accumulation while preserving physiological NO signaling [21,22].

VBI-1 is manufactured under sterile, controlled conditions to ensure reproducibility and colloidal stability. Physicochemical characterization demonstrates a mean particle diameter of approximately 17 nm, consistent with nanoscale vascular distribution. The formulation exhibits a low polydispersity index (PDI = 0.138), indicating a relatively narrow particle size distribution, and a negative zeta potential (−46 mV), consistent with electrostatic stability and reduced aggregation under physiological conditions. These parameters support the uniformity and stability of the nanoparticle system and are consistent with previously reported characterization [20].

Overall, VBI-1 functions as a phospholipid-based colloidal volume expander composed of micellar structures and phospholipids, designed not only to restore blood volume but also to redistribute nitric oxide during ischemia–reperfusion, thereby contributing to its proposed protective effects [20].

### 2.3. Experimental Design and Animals

Adult Sprague–Dawley rats were randomly assigned to one of three experimental groups: Sham, Blood, or VBI-1 (*n* = 18 total, 3 male and 3 female animals in each group). Animals were randomly assigned to experimental groups using a predefined allocation scheme to minimize selection bias. All behavioral assessments and histological analyses were performed by investigators blinded to both treatment group and sex to ensure unbiased outcome evaluation. To reduce inter-animal variability, all procedures were conducted under standardized conditions, including consistent surgical techniques, anesthesia protocols, perioperative monitoring, and environmental housing parameters across all experimental groups. All procedures were conducted in accordance with institutional guidelines for animal care and use and were approved by the Institutional Animal Care and Use Committee (IACUC).

**Figure 1:**
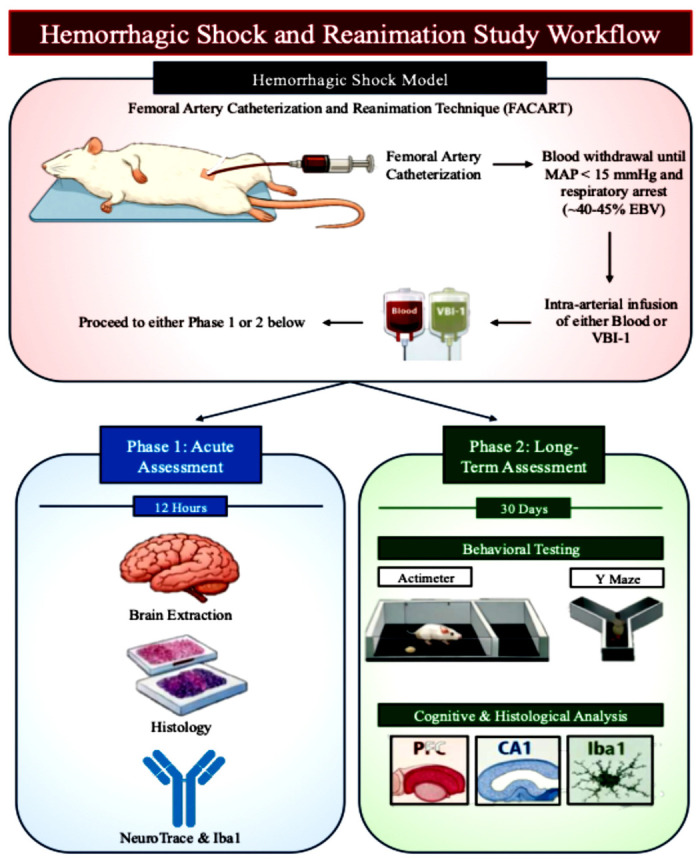
Hemorrhagic Shock and Reanimation Workflow.

The study was conducted in two phases. Phase 1 evaluated acute outcomes and tissue pathology following hemorrhagic shock-induced clinical death and reanimation over 12 h. Phase 2 assessed long-term neurological and behavioral recovery over 30 days following reanimation (Figure 1).

### 2.4. Hemorrhagic Shock-Induced Clinical Death and Reanimation (FACART Model)

To model the most severe form of hemorrhagic shock, we developed the Femoral Artery Catheterization and Reanimation Technique (FACART) to induce clinical death, defined by profound hypotension, cessation of spontaneous respiration, and loss of palpable pulses [20].

Following pre-procedural body weight measurement, animals were anesthetized with 4% isoflurane in oxygen and maintained under anesthesia throughout the surgical procedure. Animals were placed on a thermostatically controlled heating pad to maintain normothermia. Bilateral femoral vessels were surgically exposed under sterile conditions. A catheter was inserted into one femoral artery for continuous blood pressure monitoring, while the contralateral femoral artery was used for blood withdrawal.

Hemorrhagic shock was induced by controlled arterial blood withdrawal until mean arterial pressure fell below 15 mmHg, accompanied by cessation of spontaneous respiration and palpable pulses, consistent with clinical death. The volume of withdrawn blood was recorded and corresponded to approximately 40–45% of the estimated total blood volume.

Within one minute of respiratory arrest, animals were reanimated via rapid intra-arterial (IA) infusion through the femoral catheter. Animals in the Blood group received reinfusion of their blood shed, while animals in the VBI-1 group received a volume of VBI-1 equal to the volume of blood removed. Sham animals underwent identical anesthesia and femoral artery catheterization for 12 h, but did not undergo blood withdrawal or fluid infusion.

#### Acute and Chronic Experimental Phases (Phase 1 and 2)

Phase 1: Acute Injury Assessment (12 h)

In Phase 1, animals were maintained under anesthesia for 12 h following reanimation. At the conclusion of this period, animals were euthanized, and their brains were collected for histological and immunohistochemical analyses.

Phase 2: Chronic Recovery (30 days)

In Phase 2, animals were allowed to recover following reanimation and were monitored for 30 days. Behavioral testing was conducted longitudinally to assess motor activity and cognitive recovery.

### 2.5. Longitudinal Behavioral Assessment

#### 2.5.1. Actimeter Testing

Spontaneous motor activity was assessed using an automated actimeter system. Baseline activity was recorded before surgery. Post-resuscitation testing was conducted on Days 3, 7, 14, 21, and 28.

Each testing session quantified total locomotion, activity counts, and zone-specific behavior (outer vs. inner zones). Outer-zone activity was used as the primary behavioral endpoint because it is sensitive to cortico-hippocampal integrity. Data were collected for each animal and analyzed longitudinally.

#### 2.5.2. Y-Maze Spontaneous Alternation

Hippocampal-dependent working memory was assessed using the Y-maze spontaneous alternation task on Day 29 post-surgery. Animals were allowed to explore the maze freely, and arm entries were recorded. Spontaneous alternation percentage was calculated as an index of spatial working memory. Testing was conducted by investigators blinded to treatment and sex.

### 2.6. Brain Extraction and Tissue Processing

At study endpoints, animals were deeply anesthetized and transcardially perfused with phosphate-buffered saline, followed by fixative. Brains were removed, post-fixed, and cryoprotected. Coronal sections encompassing the prefrontal cortex (PFC) and hippocampal CA1 region were prepared for histological and immunohistochemical analyses.

### 2.7. Histological and Immunohistochemical Analyses

#### 2.7.1. Hematoxylin and Eosin (H&E) Staining

Sections were stained with hematoxylin and eosin to assess overall tissue architecture, neuronal morphology, neuropil integrity, and laminar organization in the PFC and hippocampal CA1. Qualitative assessment focused on neuronal density, nuclear morphology, continuity of the pyramidal layer, and neuropil vacuolation.

#### 2.7.2. NeuroTrace Staining

Neuronal soma integrity and Nissl substance were evaluated using NeuroTrace fluorescent staining. NeuroTrace-positive neurons were quantified as cells per mm^2^, and integrated fluorescence density was measured using ImageJ/FIJI (2026) to assess neuronal metabolic and translational integrity.

#### 2.7.3. Iba1 Immunohistochemistry

Microglial burden was assessed using Iba1 immunostaining. Endpoints included microglial density (cells/mm^2^), area fraction, and nearest-neighbor distance to evaluate spatial organization [26].

### 2.8. Semi-Quantitative Histological Analyses

#### 2.8.1. Cortical Neuronal Density and Laminar Organization

Neuronal density within prefrontal cortical layers II–V was quantified using standardized regions of interest (ROIs) and expressed as neurons per mm^2^, consistent with established cortical histological approaches [27]. Cortical laminar organization was assessed using a semi-quantitative layer organization index (0–3), reflecting increasing degrees of laminar disruption as previously described in ischemic brain injury models [22], reflecting preservation of cortical stratification.

#### 2.8.2. Hippocampal CA1 Neuronal Density and Degeneration Index

CA1 neuronal density was quantified by counting intact pyramidal neurons within the stratum pyramidale, consistent with established models of hippocampal ischemic injury [8,22]. Neuronal degeneration severity was assessed using a semi-quantitative degeneration index (0–4) that incorporated nuclear morphology, soma integrity, and the continuity of the pyramidal cell layer, as previously described in ischemia and traumatic brain injury models [22,28].

### 2.9. Microglial Skeletonization and Morphometric Extraction

To representatively visualize microglial process architecture, individual Iba1^+^ microglia were selected from high-magnification prefrontal cortical images using predefined criteria (intact soma, minimal overlap with neighboring cells, and complete processes within the imaging field). Images were analyzed in a standardized pipeline: the green channel was extracted, contrast-normalized using percentile-based intensity rescaling, and lightly smoothed (Gaussian filter) to reduce high-frequency noise. Binary segmentation was performed using Otsu thresholding, followed by morphological cleanup (removal of small objects, hole filling, and closing) to generate a contiguous cell mask. When multiple connected components were present, the largest component corresponding to the selected cell was retained. The binary mask was skeletonized to a one-pixel-wide representation of processes. Skeleton descriptors were extracted per cell, including total skeleton length (pixels), number of endpoints (pixels with a single 8-neighbor connection), and number of junctions (pixels with ≥3 8-neighbor connections), with optional normalization to skeleton length. Because this analysis was conducted on representative cells, results are presented descriptively and used to support qualitative interpretation of treatment-associated differences in process architecture.

### 2.10. Statistical Analyses

Given the exploratory and mechanistic nature of this study, the sample size was determined based on feasibility, prior experience with the FACART model, and consistency with our prior studies using this model [20]. Effect sizes and trajectory-based analyses were prioritized over strict hypothesis testing.

Furthermore, we emphasize the following points:

Effect sizes (Hedges’ g) and longitudinal trends were utilized to support data interpretation.

The findings are intended to inform hypothesis generation for future studies with larger sample sizes and greater statistical power.

#### 2.10.1. Longitudinal Recovery Trajectories

Longitudinal behavioral data were analyzed using linear mixed-effects models. This accounted for repeated measurements within individual animals over time. Fixed effects included time, treatment, sex, and their interactions. Animal identity was included as a random effect to account for within-subject variability. This approach was chosen to model correlated longitudinal data and handle missing observations. Model assumptions were checked using residual diagnostics.

#### 2.10.2. Planned Contrasts and Recovery Strength

Planned contrasts were performed at late recovery time points (Days 21–28). This was performed to evaluate treatment-related differences during the chronic phase. Recovery strength was defined as the change in behavioral performance between Day 3 and Day 21. This interval represents the subacute recovery period most sensitive to treatment effects. Due to limited sample size, effect size magnitude and direction (Hedges’ g) were prioritized to assess biological relevance instead of relying only on null-hypothesis significance testing.

#### 2.10.3. Sex-Stratified Cognitive and Histological Analyses

Histological and cognitive outcomes were analyzed using two-way analysis of variance (ANOVA). Treatment and sex were used as fixed factors. When significant main effects or interactions were found, post hoc comparisons were performed with appropriate multiple comparison corrections. Assumptions of normality and homogeneity of variance were checked. Analyses were interpreted with sample size limitations in mind.

#### 2.10.4. Structure–Function Correlations

Associations between neuronal integrity (e.g., neuronal density and NeuroTrace intensity) and behavioral performance were assessed using Spearman’s rank correlation at the individual-animal level. This non-parametric approach was chosen because histological measures are semi-quantitative and data might not be normally distributed. Correlation strength and direction were used to evaluate structure–function relationships.

Statistical significance was set at *p* < 0.05. Data are presented as mean ± SEM.

## 3. Results

### 3.1. Longitudinal Behavioral Recovery Trajectories Following Blood Injury and VBI-1 Treatment

Across all behavioral outcomes, a significant main effect of time was observed, indicating dynamic changes in behavior throughout the post-surgical recovery period (linear mixed-effects model: F(2.868, 37.29) = 7.419, *p* = 0.0006). These findings suggest progressive behavioral recovery from the acute to the chronic phase.

Further analysis using linear mixed-effects modeling indicated that recovery trajectories varied by treatment and sex, with the most pronounced effects observed in outer-zone behaviors, which are sensitive to cortico-hippocampal function.

#### 3.1.1. Treatment- and Sex-Dependent Recovery in Outer-Zone Activity and Locomotion

**Figure 2:**
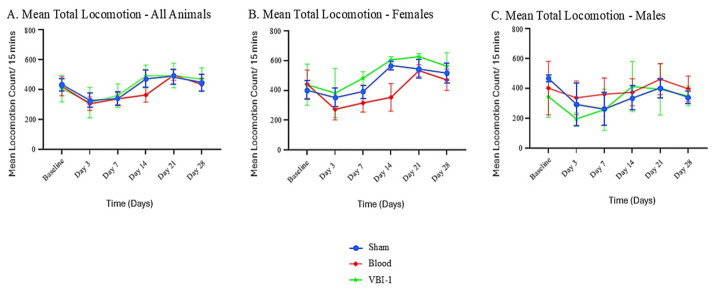
Longitudinal behavioral recovery trajectories and recovery strength (Days 1–28). (**A**) Mean total locomotion across all animals, independent of sex. (**B**) Mean total locomotion in female animals. (**C**) Mean total locomotion in male animals. Data are presented as mean ± SEM. Behavioral recovery exhibits significant time-dependent changes, with treatment- and sex-specific differences emerging during the late recovery phase.

In the outer zone, both activity and locomotion increased progressively from the Trial time point through Day 28. Mixed-effects analysis revealed significant main effects of time (F(5, 36) = 3.693, *p* = 0.0084) and treatment (F(2, 36) = 3.442, *p* = 0.0429), supporting the presence of differential recovery profiles across experimental groups (Figure 2).

Sex-stratified analyses suggest distinct recovery patterns. Female rats treated with VBI-1 exhibited sustained improvement during the late recovery phase, particularly between Days 21 and 28. In contrast, male rats appeared to show more rapid early recovery following whole-blood resuscitation, with performance stabilizing at later time points. These observations are consistent with sex-dependent differences in treatment response, rather than uniform behavioral recovery across groups (Figure 2).

#### 3.1.2. Planned Contrasts at Late Recovery Time Points (Days 21–28)

Planned contrasts were conducted at late recovery time points (Days 21–28) to assess whether VBI-1 approached or exceeded the efficacy of whole-blood resuscitation during the chronic phase of recovery. Given the limited sample size, effect size magnitude and direction (Hedges’ g) were prioritized over null-hypothesis significance testing to assess biologically meaningful differences.

In female rats, resuscitation with VBI-1 was associated with recovery of outer-zone behavioral endpoints that were comparable to or greater than those observed with whole blood, including activity, locomotion, and rearing. These differences were supported by large to very large positive effect sizes, suggesting a potential advantage of VBI-1 during late recovery (Table 1).

In contrast, male rats showed a trend toward greater late-phase recovery following whole-blood resuscitation than VBI-1-treated males, although VBI-1-treated males still showed improvement relative to sham controls. Effect sizes consistently favored blood over VBI-1 in males, with moderate-to-large magnitudes across behavioral measures (Table 2). Together, these findings suggest that treatment efficacy during late recovery may be sex-dependent, with VBI-1 conferring a relative advantage in females.

#### 3.1.3. Recovery Strength During the Subacute Phase (Day 3–Day 21)

Quantification of recovery strength identified the Day 3-Day 21 interval as a sensitive window for detecting treatment-related differences. In female rats, VBI-1 was associated with greater recovery strength across outer-zone behavioral measures compared with whole blood, with consistently large effect sizes, supporting a biologically meaningful enhancement during this subacute period (Table 1).

In male rats, whole blood appeared to support stronger recovery over the same interval; however, VBI-1 still promoted functional improvement relative to sham treatment. These findings are consistent with sex-dependent recovery patterns, rather than differences driven solely by baseline activity or late-stage stabilization (Table 2).

### 3.2. Behavioral Safety Assessment

Across all time points and treatment groups, no increases in stereotypic behavior or inner-zone activity were observed. These findings suggest that neither VBI-1 nor whole blood exacerbated repetitive or anxiety-like behaviors, supporting the behavioral safety profile of VBI-1 as a resuscitation fluid.

#### 3.2.1. Integrated Interpretation

Collectively, these results suggest that VBI-1 supports sustained behavioral recovery following hemorrhagic shock, with recovery trajectories that are both time-dependent and sex-dependent. In females, VBI-1 is associated with recovery comparable to or exceeding that of whole blood, particularly during the subacute-to-chronic phase. In contrast, whole blood appears to confer a relative advantage in males during certain recovery intervals. Importantly, these functional observations occur without evidence of adverse behavioral effects, supporting the potential therapeutic relevance of VBI-1 while warranting cautious interpretation.

#### 3.2.2. Sex-Dependent Effects on Hippocampal-Dependent Working Memory (Y-Maze)

**Figure 3:**
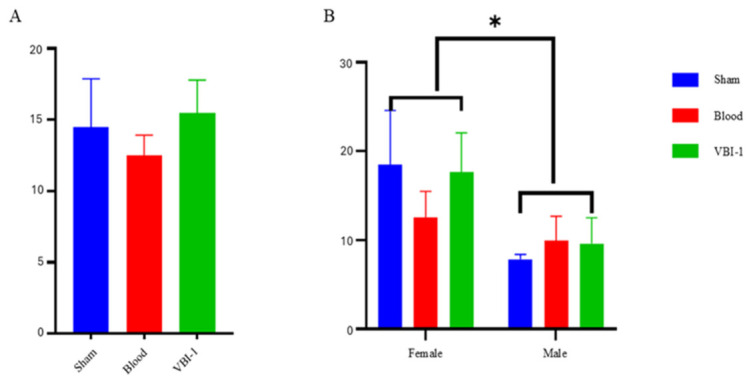
Sex-dependent effects of blood injury and VBI-1 on Y-maze spontaneous alternation. (**A**) Overall percentage spontaneous alternation across Sham, Blood, and VBI-1 groups, independent of sex. Behavioral performance shows modest variation across treatment groups, with no clear separation at the aggregate level. (**B**) Sex-stratified spontaneous alternation in female and male animals. Females exhibit higher baseline alternation in the Sham group, a reduction following blood injury, and recovery toward sham levels following VBI-1 treatment. In contrast, males show lower baseline alternation and comparatively limited changes across treatment conditions. Data are presented as mean ± SEM. Statistical comparisons are indicated by brackets; * *p* < 0.05. A significant Sex × Treatment interaction was observed, indicating that the effect of VBI-1 differs between females and males.

Analysis of spontaneous alternation behavior revealed sex-dependent effects of blood injury and VBI-1 treatment on spatial working memory (Figure 3).

In the Sham group, females exhibited higher baseline alternation rates than males, suggesting a baseline sex difference in hippocampal-dependent cognition. Blood injury resulted in reduced alternation performance in both sexes; however, the magnitude and pattern of impairment differed. Female animals showed a marked reduction relative to sham, whereas males exhibited persistently low and variable performance, consistent with a potential floor effect.

Resuscitation with VBI-1 was associated with a sex-dependent pattern of cognitive recovery. Female animals treated with VBI-1 showed increased spontaneous alternation compared with the Blood group, approaching sham levels. In contrast, male animals treated with VBI-1 showed minimal and variable improvement, with performance remaining similar to that of the Blood group.

Two-way ANOVA (Treatment × Sex) revealed a main effect of sex (F(1, 12) = 5.529, *p* = 0.0366), with females exhibiting higher alternation overall, as well as a main effect of treatment. A Sex × Treatment interaction was observed, supporting the interpretation that treatment effects differ by sex. Post hoc comparisons indicated that VBI-1 improved spontaneous alternation in females relative to the Blood group.

Taken together, these findings suggest that VBI-1 may preferentially support hippocampal-dependent working memory recovery in females, whereas males exhibit more limited cognitive improvement under the conditions tested.

Two-way ANOVA (Treatment × Sex) revealed a main effect of sex; F (1, 12) = 5.529, *p* = 0.0366, with females exhibiting higher alternation percentages overall, and a main effect of treatment. Importantly, a Sex × Treatment interaction was observed, indicating that the cognitive effects of VBI-1 differed between females and males. Post hoc comparisons showed that VBI-1 improved spontaneous alternation in females compared with the Blood group.

These data indicate that VBI-1 selectively improves hippocampal-dependent working memory in females, whereas males exhibit limited behavioral recovery.

### 3.3. Sex-Specific Effects of Blood Injury and VBI-1 on Prefrontal Cortical Integrity

**Figure 4:**
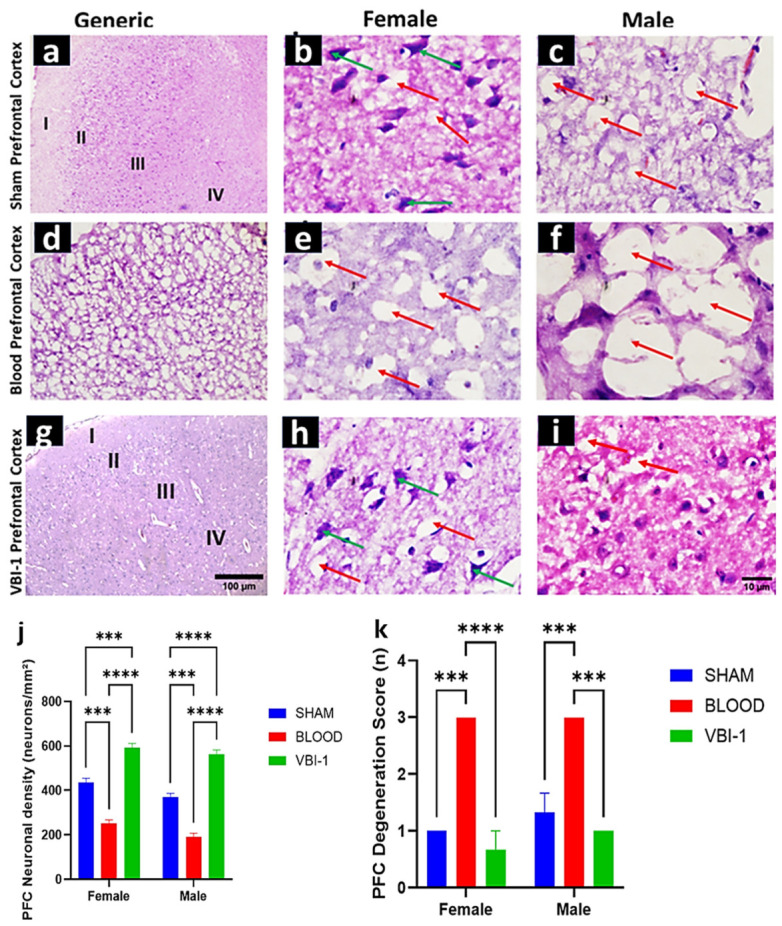
Sex-specific effects of blood injury and VBI-1 on prefrontal cortical architecture (**a**–**i**)**.** Representative H&E-stained sections of the prefrontal cortex from female (**b**,**e**,**h**) and male (**c**,**f**,**i**) animals in the Sham, Blood, and VBI-1 groups. Blood injury induces severe neuropil vacuolation, neuronal loss, and laminar disorganization, with greater pathology observed in males. VBI-1 treatment markedly preserves neuronal density and cortical lamination, with near-complete rescue in females. Scale bar = 100 µm [(**a**–**c**); Mag = x100], 10 µm [(**b**,**c**,**e**,**f**,**h**,**i**); Mag = ×400]. Figures (**j**) and (**k**): Semi-quantitative analysis of cortical neuronal density and laminar organization, respectively. Bar graphs showing cortical neuronal density (neurons/mm^2^, layers II–V) and layer organization index, stratified by sex. Data represent mean ± SEM. *** *p* < 0.001, **** *p* < 0.0001. Two-way ANOVA demonstrates significant effects of treatment and sex, with females exhibiting greater VBI-1-mediated neuroprotection.

Representative H&E-stained sections of the prefrontal cortex (PFC) revealed sex-dependent differences in cortical pathology following blood injury and VBI-1 treatment (Figure 4k).

In the Blood group, both female and male animals exhibited diffuse cortical alterations characterized by neuropil vacuolation (red arrows), reduced neuronal density, and disruption of laminar organization across layers II–V. These pathological features were more pronounced in males, who showed greater extracellular rarefaction, fewer identifiable neuronal somata, and more extensive loss of laminar boundaries than females.

In contrast, Sham animals of both sexes displayed preserved cortical cytoarchitecture, with dense neuropil, clearly identifiable neuronal somata (green arrows), and intact laminar organization. Subtle sex-related differences were observed, with sham females exhibiting modestly higher neuronal density and more defined cortical layering than sham males.

Treatment with VBI-1 was associated with attenuation of blood-induced cortical pathology in both sexes. VBI-1-treated females exhibited preservation of cortical architecture, reduced vacuolation, and well-defined neuronal somata, with features approaching those observed in sham animals. VBI-1-treated males also demonstrated structural improvement relative to the Blood group; however, mild residual neuropil vacuolation and subtle laminar disruption persisted, suggesting partial recovery compared with females.

#### Semi-Quantitative Analysis of Cortical Neuronal Density and Laminar Organization

Cortical neuronal density within layers II–V was semi-quantified using standardized regions of interest and expressed as neurons per mm^2^. Laminar organization was assessed using a qualitative layer organization index (0–3).

Two-way ANOVA (Treatment × Sex) revealed a significant main effect of treatment on cortical neuronal density and laminar organization (*p* < 0.001 for both outcomes). A significant main effect of sex was also observed, with males exhibiting greater cortical pathology than females across treatment conditions (*p* < 0.05). A significant Treatment × Sex interaction (*p* < 0.05) suggested that the magnitude of injury and the response to VBI-1 differed between sexes (Figure 4j).

Post hoc analyses indicated that VBI-1 was associated with improved neuronal density and laminar organization relative to the Blood group in both females and males (*p* < 0.01), with larger effect sizes observed in females. Blood injury resulted in a substantial reduction in cortical neuronal density in both sexes; however, males exhibited a greater magnitude of neuronal loss (approximately 60–65% reduction relative to sham) compared with females (approximately 45–50% reduction). Sham females displayed modestly higher baseline neuronal density than sham males. VBI-1 treatment was associated with restoration of neuronal density in both sexes, with values in females approaching sham levels and a substantial, though comparatively attenuated, recovery observed in males.

### 3.4. Sex-Specific Effects of Blood Injury and VBI-1 on Hippocampal CA1 Neuronal Integrity

**Figure 5:**
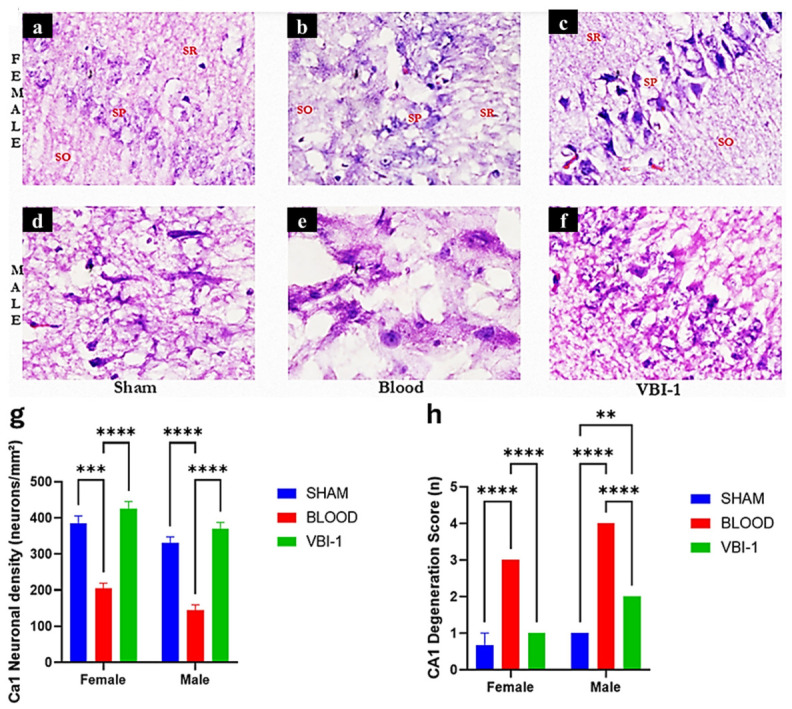
Sex-specific effects of blood injury and VBI-1 on hippocampal CA1 neuronal integrity. Representative high-magnification hematoxylin and eosin (H&E)-stained sections of the hippocampal CA1 region from female (**a**–**c**) and male (**d**–**f**) animals in the Sham, Blood, and VBI-1 groups. Blood injury induces marked pyramidal neuron degeneration, characterized by neuronal shrinkage, hyperchromatic nuclei, disruption of the stratum pyramidale (SP), and pronounced neuropil rarefaction within the stratum oriens (SO) and stratum radiatum (SR), with greater severity observed in males. In contrast, VBI-1 treatment preserves neuronal morphology, maintains pyramidal layer organization, and reduces neuropil disruption, with near-complete structural rescue in females and partial recovery in males. Quantitative analysis (**g**,**h**) shows CA1 neuronal density (neurons/mm^2^) and degeneration index across groups, demonstrating a significant reduction in neuronal density and increased degeneration following blood injury, with a greater magnitude of injury in males. VBI-1 treatment significantly improves neuronal density and reduces degeneration scores in both sexes, with a more pronounced protective effect in females. Data are presented as mean ± SEM. Statistical significance was determined using two-way ANOVA (treatment × sex) followed by appropriate post hoc comparisons. ** *p* < 0.01, *** *p* < 0.001, **** *p* < 0.0001. Scale bar = 50 µm; magnification = ×400.

Representative high-magnification H&E-stained sections of the hippocampal CA1 region revealed pronounced treatment- and sex-dependent alterations in pyramidal neuron morphology and neuropil integrity (Figure 5).

In the Blood group, CA1 pyramidal neurons exhibited degenerative changes in both sexes, including neuronal shrinkage, hyperchromatic and pyknotic nuclei, and disruption of the pyramidal cell layer. Neuropil vacuolation and extracellular clearing were observed throughout the stratum pyramidale (SP) and adjacent regions, including the stratum oriens (SO) and stratum radiatum (SR).

These pathological features were more pronounced in males, who showed a higher frequency of pyknotic neurons, greater disruption of pyramidal alignment, and greater neuropil rarefaction than females.

In contrast, Sham animals of both sexes displayed preserved CA1 architecture, characterized by densely packed pyramidal neurons with round-to-oval euchromatic nuclei and intact laminar organization. Subtle differences were observed: females exhibited slightly higher neuronal density and more uniform pyramidal alignment than males.

Treatment with VBI-1 was associated with attenuation of CA1 pathology in both females and males. VBI-1-treated females demonstrated preservation of pyramidal neuron morphology, reduced frequency of pyknotic nuclei, and improved continuity of the pyramidal layer, with features approaching those observed in sham animals. VBI-1-treated males also exhibited structural improvement relative to the Blood group; however, mild residual neuronal shrinkage and focal neuropil vacuolation persisted, suggesting partial recovery compared with females.

#### Semi-Quantitative CA1 Neuronal Density and Degeneration Index

CA1 neuronal density was semi-quantified by counting intact pyramidal neurons within standardized regions of the stratum pyramidale and expressed as neurons per mm^2^. Degeneration severity was assessed using a semi-quantitative degeneration score (0–4) that incorporated nuclear morphology, soma integrity, and pyramidal layer continuity.

Blood injury was associated with a substantial reduction in CA1 neuronal density in both sexes; however, males exhibited a greater magnitude of neuronal loss (approximately 60–65% reduction relative to sham) than females (approximately 45–50%). Sham females demonstrated modestly higher baseline neuronal density than sham males.

VBI-1 treatment was associated with preservation of CA1 neuronal density in both sexes, with values in females approaching sham levels and substantial, though comparatively attenuated, recovery observed in males. Degeneration scores followed a similar pattern, with blood injury associated with increased degeneration severity in both sexes, particularly in males. VBI-1 treatment was associated with reduced degeneration scores, with a greater magnitude of improvement observed in females.

### 3.5. Integration with CA1 and Prefrontal NeuroTrace Pathology

The sex-dependent behavioral effects observed in the Y-maze closely parallel histopathological alterations within the hippocampal CA1 region, a structure critically involved in spatial working memory. Female animals treated with VBI-1 exhibited greater preservation of CA1 pyramidal neuron density and reduced degenerative changes compared with males. In contrast, blood-resuscitated males exhibited pronounced neuronal loss, disruption of the pyramidal layer, and incomplete histological recovery. These CA1 findings align with superior spontaneous alternation performance in females and persistently impaired performance in males, supporting a hippocampal contribution to the observed sex-dependent cognitive outcomes.

**Figure 6:**
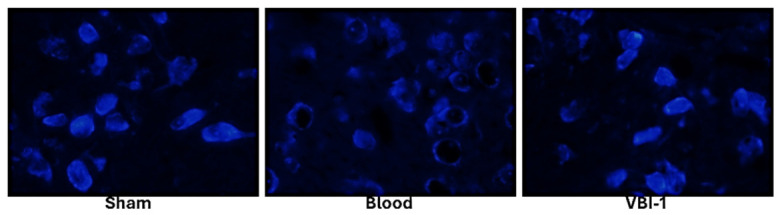
NeuroTrace (Nissl) staining demonstrates treatment-dependent preservation of neuronal soma integrity in the prefrontal cortex. Representative NeuroTrace-labeled sections from Sham**,** Blood, and VBI-1 groups show neuronal cell bodies with intact Nissl substance (bright blue fluorescence) within the prefrontal cortex. Blood animals exhibit visibly reduced NeuroTrace-positive neuronal profiles and weaker/patchier staining consistent with impaired neuronal soma integrity and disrupted neuropil architecture, whereas VBI-1 shows relative preservation of NeuroTrace signal and neuronal profiles compared with Blood, approaching the organization seen in Sham. Images are representative of the cohort and were acquired using an identical microscope and exposure settings across groups. Quantification (reported in Results) was performed as NeuroTrace-positive neuron density (cells/mm^2^) and NeuroTrace integrated fluorescence density using standardized ROIs (layers II–V) and uniform thresholding in ImageJ/FIJI.

Importantly, assessment of neuronal soma integrity using NeuroTrace staining was performed in the prefrontal cortex (PFC), providing complementary insight into cortical neuronal functional status. NeuroTrace labeling, which reflects preservation of Nissl substance and neuronal translational capacity, was markedly better preserved in the PFC of VBI-1-treated compared with blood-resuscitated animals (Figure 6). This pattern suggests that VBI-1 preferentially preserves cortical neuronal metabolic competence, consistent with improved executive and exploratory behaviors observed during late recovery.

Together, these findings support a distributed structure–function model in which recovery of hippocampal-dependent working memory is associated with preservation of CA1 neuronal integrity, while sustained behavioral and executive recovery is further supported by maintenance of prefrontal cortical neuronal soma integrity, as reflected by NeuroTrace staining [29]. By integrating region-specific histopathological markers, CA1 neuronal density and degeneration with PFC NeuroTrace integrity, these data demonstrate that VBI-1-mediated neuroprotection operates across multiple nodes of the cortico-hippocampal network. This multi-level preservation of neuronal structure and function provides a mechanistic basis for the observed sex-dependent differences in cognitive and behavioral recovery following hemorrhagic shock-induced clinical death.

### 3.6. Neuronal Density Is a Meaningful Predictor of Cognitive Outcome 

**Figure 7:**
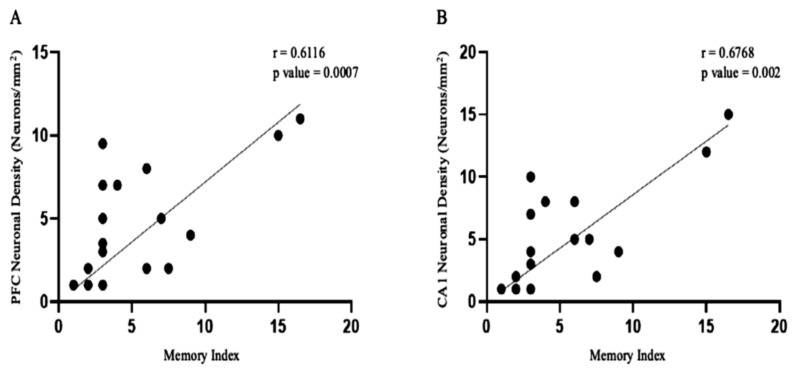
Structural–functional correlation between neuronal density and hippocampal-dependent memory performance. (**A**) Scatter plot showing the relationship between prefrontal cortical neuronal density and outer-zone behavioral activity across all animals. Each point represents an individual animal; a positive association suggests that higher neuronal density is associated with improved behavioral performance. (**B**) Scatter plot illustrates the relationship between hippocampal CA1 neuronal density and Y-maze spontaneous alternation performance. Higher neuronal density is associated with improved spatial working memory. Spearman correlation coefficients (r) and corresponding p-values are indicated where applicable.

To determine whether preservation of neuronal architecture translated into functional cognitive recovery, animal-level correlations were performed between neuronal density and hippocampal-dependent working memory performance. Given the semi-quantitative nature of the histological measures and the non-Gaussian distribution of behavioral data, Spearman’s rank correlation was used (Figure 7).

Across all animals, neuronal density was positively correlated with memory performance, indicating that animals with greater preservation of neuronal populations exhibited superior spontaneous alternation behavior (Figure 7). This relationship supports a direct structure–function coupling between neuronal integrity and hippocampal-dependent cognition.

Sex-stratified exploratory analyses revealed that the strength of this correlation was greater in females than in males. Female animals demonstrated a clear monotonic relationship between neuronal density and memory index, consistent with the more complete histological preservation and behavioral recovery observed following VBI-1 treatment. In contrast, males exhibited a weaker and more variable association, reflecting greater residual neuronal loss and limited behavioral recovery despite treatment.

Importantly, animals treated with VBI-1 clustered toward higher neuronal density and higher memory performance, whereas blood-resuscitated animals clustered toward lower values on both axes. Sham animals occupied an intermediate-to-high range, consistent with preserved baseline structure and function.

These findings demonstrate that neuronal density is a meaningful predictor of cognitive outcome and provide quantitative support for the hypothesis that VBI-1-mediated preservation of neuronal architecture underlies functional recovery, particularly in females.

### 3.7. Microglial Burden Is Not Increased by VBI-1 Relative to Blood Resuscitation

Skeleton-based morphometric descriptors (total skeleton length, endpoints, and junctions) extracted from these representative cells were directionally consistent with the observed morphological differences and are provided as descriptive analyses.

**Figure 8:**
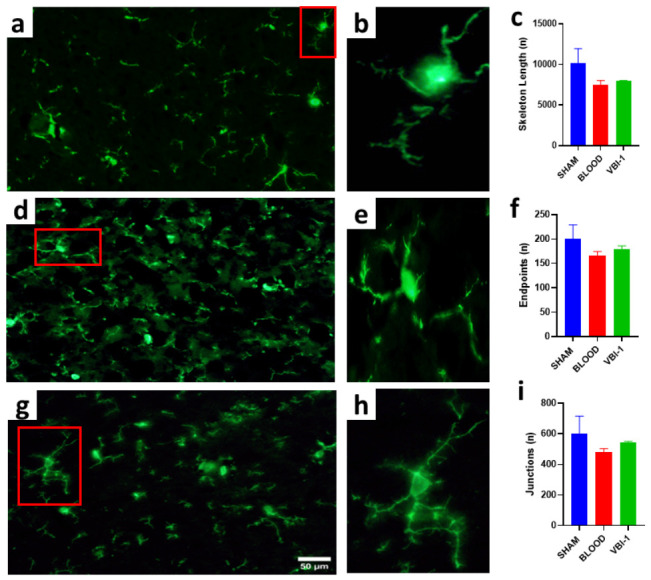
Microglial activation in the rat prefrontal cortex (PFC): Representative immunohistochemical images showing microglial labeling using ionized calcium-binding adaptor molecule-1 (Iba1) in the prefrontal cortex of rats from the sham (**a**,**b**), Blood (**d**,**e**), and VBI-1 (**g**,**h**) experimental groups; the red frames in (**a**,**d**,**g**) are the insets images for (**b**,**e**,**h**) respectively. Iba1-positive microglia demonstrate group-dependent differences in morphology and distribution within the PFC. Skeletonization was applied to binarized images to visualize microglial process architecture over the original Iba1 signal. Representative cells were selected based on intact soma, minimal overlap with neighboring cells, and complete processes within the imaging field. Compared with sham, whole blood resuscitation was associated with reduced ramification and shortened processes, whereas VBI-1 preserved a more highly ramified morphology, consistent with a surveillant microglial state (**c**,**f**,**i**). Images were acquired at 40× magnification. Scale bar = 50 µm.

To determine whether VBI-1 alters neuroinflammatory responses relative to blood resuscitation, microglial burden was assessed using Iba1 immunoreactivity across multiple complementary endpoints, including microglial density, area fraction, and spatial organization (Figure 8).

#### 3.7.1. Microglial Burden and Spatial Organization Following Blood Injury and VBI-1 Treatment

Representative Iba1 immunostaining and quantitative analyses of microglial density, area fraction, and nearest-neighbor distance in female and male animals across Sham, Blood, and VBI-1 groups. No significant differences were observed between VBI-1 and blood resuscitation for any microglial endpoint, indicating that VBI-1 does not exacerbate microglial burden or disrupt microglial spatial organization. Data represent mean ± SEM.

Animal-level analysis of Iba1-positive microglial density revealed no significant main effect of treatment, no main effect of sex, and no treatment × sex interaction (two-way ANOVA; all *p* > 0.5). Importantly, planned contrasts demonstrated that VBI-1-treated animals did not exhibit increased microglial density compared with blood-resuscitated animals in either females or males (both *p* > 0.4).

#### 3.7.2. Iba1 Area Fraction

**Figure 9:**
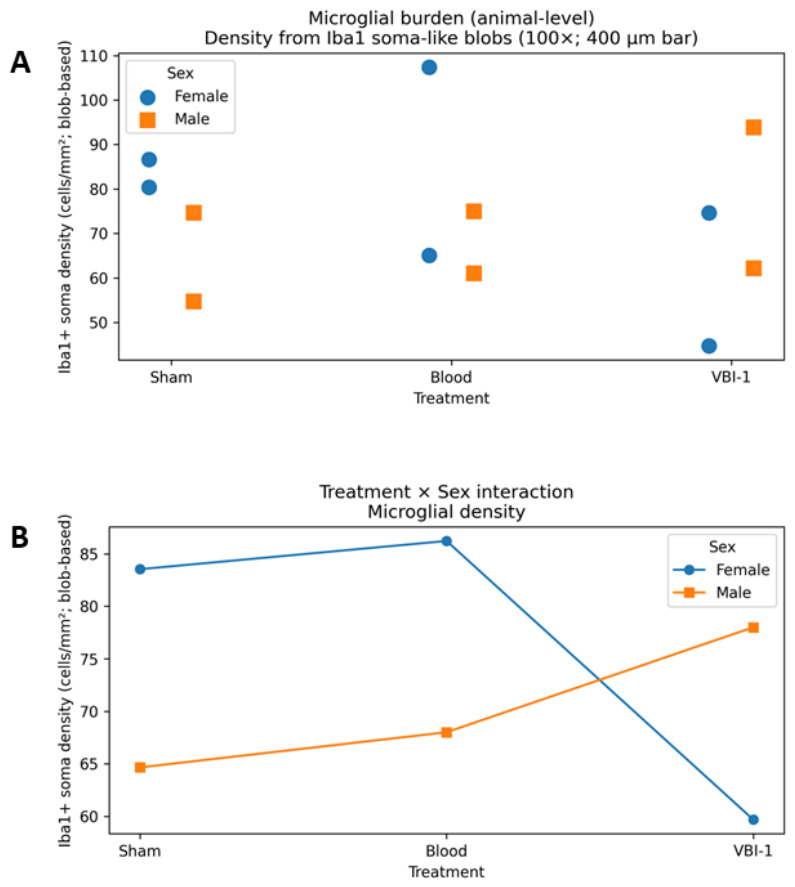
Microglial density. (**A**) Animal-level quantification of Iba1-positive soma density in the prefrontal cortex across Sham, Blood, and VBI-1 groups. Each point represents an individual animal, with females and males shown separately. Iba1-positive soma density was expressed as cells/mm^2^ using blob-based soma detection from 100× images with a 400 µm scale reference. (**B**) Treatment × Sex interaction plot for microglial density. Females showed higher Iba1-positive soma density in the Sham and Blood groups, followed by lower density in the VBI-1 group, whereas males showed a gradual increase across Sham, Blood, and VBI-1 groups. This pattern suggests a sex-dependent difference in microglial burden following VBI-1 treatment. Data are presented as animal-level values, and group means by sex.

Consistent with density findings, Iba1 area fraction, a measure of overall microglial coverage within the tissue, did not differ significantly by treatment, sex, or their interaction (all *p* > 0.7). Direct within-sex comparisons confirmed that VBI-1 did not increase microglial coverage relative to blood in either females or males (Figure 9A).

#### 3.7.3. Microglial Spatial Organization

To assess whether treatment influenced microglial territorial organization, the nearest-neighbor distance between Iba1-positive microglial somata was quantified (Figure 9B). Analysis revealed no significant effects of treatment, sex, or treatment × sex interaction (all *p* > 0.6), indicating that microglial spatial distribution remained intact following VBI-1 resuscitation and was comparable to that observed after blood resuscitation.

#### 3.7.4. Integrated Interpretation

Across all microglial endpoints—density, coverage, and spatial organization, VBI-1 resuscitation produced microglial responses indistinguishable from blood, with no evidence of exaggerated neuroinflammatory burden or sex-specific dysregulation. Sham animals exhibited variability across measures and were therefore used as a contextual reference rather than a fixed biological baseline.

Together, these findings demonstrate that VBI-1-mediated neuroprotection and behavioral recovery occur without an associated increase in microglial accumulation or disruption of microglial organization, supporting a favorable neuroinflammatory safety profile.

While the present findings suggest consistent sex-dependent differences in behavioral and histological recovery following VBI-1 resuscitation, they should be interpreted with appropriate caution. The relatively small sample size, particularly within sex-stratified analyses, limits statistical power and may reduce sensitivity to detect more subtle treatment effects. Nonetheless, the consistency of effect-size patterns and recovery trajectories across multiple behavioral and histological endpoints supports the biological relevance of these observations. In addition, although multiple microglial endpoints were assessed, the evaluation of neuroinflammation remains limited; incorporation of additional markers, such as cytokine profiling and astrocytic responses, would provide a more comprehensive characterization of the inflammatory landscape. Future studies with larger, adequately powered cohorts and expanded neuroinflammatory profiling will be important to confirm these findings and further delineate sex-specific responses to phospholipid nanoparticle-based resuscitation.

## 4. Discussion

This study demonstrates that neurological outcome following hemorrhagic shock severe enough to induce clinical death is jointly influenced by biological sex and resuscitation strategy. Using the FACART paradigm, animals were subjected to Clinical death, defined by a reduction in mean arterial pressure below 15 mmHg, loss of spontaneous respiration, and palpable pulses, followed by emergent intra-arterial reanimation. This level of insult closely recapitulates catastrophic hemorrhage encountered in civilian and battlefield settings and produces a state of global cerebral ischemia followed by reperfusion, a pathophysiological condition distinct from moderate hypovolemia models that fail to capture delayed ischemia–reperfusion brain injury [8,21,22]. By applying this stringent paradigm, the present work directly evaluates whether resuscitation strategies can preserve neurological structure and function following the most severe forms of hemorrhagic shock.

These findings extend our prior demonstration that VBI-1 restores hemodynamics, improves organ perfusion, and reduces oxidative DNA damage following hemorrhagic shock-induced clinical death [20]. Consistent with the premise that survival alone is an incomplete metric of resuscitation success, the current data indicate that the systemic benefits of VBI-1 translate into durable preservation of brain structure and function. Longitudinal behavioral analyses revealed a robust effect of time, reflecting gradual recovery after global ischemic injury; however, recovery trajectories diverged by sex and treatment. Behaviors sensitive to cortico-hippocampal integrity exhibited sustained late-phase improvement in females treated with VBI-1, whereas males showed more rapid early recovery following whole-blood resuscitation that subsequently plateaued. These divergent trajectories underscore that resuscitation efficacy cannot be assumed to be uniform across biological contexts.

Sex differences in outcome following hemorrhagic shock and global ischemia are well documented and were anticipated based on mechanisms summarized in the Introduction, including sex-dependent regulation of mitochondrial function, antioxidant capacity, and inflammatory signaling [30,31,32]. Estrogen-dependent mechanisms—such as improved cerebral blood flow regulation, reduced lipid peroxidation, and enhanced mitochondrial resilience—have been shown to confer neuroprotection following ischemic and traumatic insults [33,34,35]. The present findings are consistent with this literature and suggest that VBI-1 may engage or amplify female-specific protective pathways, resulting in superior subacute-to-chronic behavioral recovery. In contrast, the relatively early advantage of whole blood in males highlights that conventional resuscitation may transiently support recovery through oxygen-carrying capacity yet fail to prevent delayed secondary injury, a limitation of blood-based resuscitation strategies.

The hippocampal CA1 region represents a canonical site of delayed neuronal vulnerability following global ischemia [8,22]. In the present study, whole-blood-resuscitated animals exhibited marked CA1 degeneration, with greater neuronal loss and pyramidal layer disruption in males, paralleling deficits in hippocampal-dependent working memory. In contrast, VBI-1 preserved CA1 neuronal integrity, with near-complete protection in females and partial preservation in males. Importantly, CA1 neuronal density correlated strongly with spontaneous alternation performance, reinforcing the structure–function relationship between CA1 preservation and cognition [36]. These findings support the central premise articulated in the Introduction: neuronal preservation following reanimation from clinical death is a key determinant of cognitive recovery rather than a secondary correlate of survival.

Beyond the hippocampus, hemorrhagic shock-induced clinical death produced pronounced prefrontal cortical pathology, including neuronal loss, neuropil vacuolation, and laminar disorganization. The prefrontal cortex is particularly susceptible to hypoperfusion- and reperfusion-associated microvascular and metabolic injury, contributing to long-term executive dysfunction after circulatory arrest and severe shock [6]. The observed cortical pathology is consistent with prior reports linking ischemia–reperfusion injury to disruption of cortical microcircuits and altered neuromodulatory signaling [28,37]. VBI-1 markedly preserved prefrontal neuronal density and laminar organization, most prominently in females, and cortical preservation correlated with behavioral outcomes. These findings extend earlier systemic protection data for VBI-1 by demonstrating preservation of higher-order cortical networks essential for exploration and executive behaviors.

NeuroTrace labeling provided mechanistic insight by distinguishing neuronal survival from neuronal functional competence. Loss of Nissl substance following whole-blood resuscitation reflects chromatolysis and impaired protein synthesis in neurons that survive ischemia but remain metabolically compromised [38,39]. Preservation of NeuroTrace labeling with VBI-1, particularly in females, indicates maintenance of neuronal translational capacity and soma integrity. When integrated with histological and behavioral outcomes, these findings support a multi-level structure–function framework in which VBI-1 preserves neuronal density, sustains cellular metabolic competence, and maintains circuit-level function. Correlations between neuronal integrity and cognitive performance in both CA1 and prefrontal cortex further indicate that VBI-1-mediated neuroprotection is functionally meaningful rather than purely anatomical.

Reanimation following clinical death is typically accompanied by robust neuroinflammatory activation that contributes to delayed neuronal loss and cognitive decline after ischemia–reperfusion injury [40,41]. Notably, resuscitation without increasing microglial density, coverage, or spatial organization, suggesting that VBI-1 does not exacerbate the neuroinflammatory burden associated with reperfusion injury. This finding is particularly relevant given that blood-based resuscitation can amplify oxidative stress and inflammatory signaling during reperfusion. Consistent with these quantitative outcomes, representative skeleton overlays illustrate preserved microglial process architecture following VBI-1 resuscitation. Together with our prior demonstration that VBI-1 reduces oxidative DNA damage and modulates nitric oxide dynamics following hemorrhagic shock [20], the present data support the conclusion that VBI-1 confers central nervous system protection without provoking maladaptive neuroinflammation. While the observed neuroprotective effects are consistent with mechanisms such as membrane stabilization, modulating nitric oxide-mediated microvascular signaling during reperfusion, and oxidative stress, these pathways were not directly interrogated in the present study.

## 5. Conclusions

By modeling hemorrhagic shock severe enough to induce clinical death rather than survivable hypotension, this study provides a clinically relevant framework for evaluating the neuroprotective efficacy of resuscitation strategies under the most extreme conditions of circulatory collapse. The findings demonstrate that resuscitation with VBI-1 supports sustained neurological recovery following reanimation, with sex-dependent effects that merit further investigation in both civilian trauma systems and military medicine. The relatively small sample size, particularly within sex-stratified analyses, limits statistical power and necessitates cautious interpretation. While consistent effect size patterns support biological relevance, these findings should be validated in larger, adequately powered studies.

Our proprietary phospholipid nanoparticle represents a promising next-generation resuscitation strategy that can improve neurological outcomes following severe hemorrhagic shock. Importantly, VBI-1 shifts the therapeutic goal of hemorrhagic shock management beyond survival alone toward meaningful long-term recovery. By preserving neuronal structure and functional integrity in vulnerable brain regions without exacerbating neuroinflammation, VBI-1 offers a novel approach to preventing secondary brain injury after profound ischemia–reperfusion events.

## Figures and Tables

**Table 1:** Late-Phase Recovery Strength in Females: VBI-1 vs. Whole Blood (Days 21–28).

Metric	Effect Size (Hedges’ g)	Interpretation
Activity	Large (≈ +0.8 to +1.2)	VBI-1 > Blood
Locomotion	Large (≈ +0.9 to +1.3)	VBI-1 > Blood
Rearing	Very large (≈ +1.2 to +1.6)	VBI-1 >> Blood

Interpretation: In females, VBI-1 produces substantially stronger late-phase recovery than whole blood, particularly for exploratory and locomotor behaviors.

**Table 2:** Late-Phase Recovery Strength in Males: Whole Blood vs. VBI-1 (Days 21–28).

Metric	Effect Size (Hedges’ g)	Interpretation
Activity	Moderate–large (≈ −0.6 to −1.0)	Blood > VBI-1
Locomotion	Large (≈ −0.8 to −1.2)	Blood > VBI-1
Rearing	Moderate (≈ −0.5 to −0.8)	Blood > VBI-1

Negative effect sizes indicate greater recovery following blood resuscitation relative to VBI-1.

## Data Availability

The original contributions presented in this study are included in the article. Further inquiries can be directed to the corresponding authors.
